# A Novel Homozygous Mutation in the EC1/EC2 Interaction Domain of the Gap Junction Complex Connexon 26 Leads to Profound Hearing Impairment

**DOI:** 10.1155/2014/307976

**Published:** 2014-01-16

**Authors:** Ralf Birkenhäger, Nicola Prera, Antje Aschendorff, Roland Laszig, Susan Arndt

**Affiliations:** Department of Otorhinolaryngology, Head and Neck Surgery, University Medical Center Freiburg, Killianstraße 5, 79106 Freiburg, Germany

## Abstract

To date, about 165 genetic loci or genes have been identified which are associated with nonsyndromal hearing impairment. In about half the cases, genetic defects in the *GJB2* gene (connexin 26) are the most common cause of inner-ear deafness. The genes *GJB2* and *GJB6* are localized on chromosome 13q11-12 in tandem orientation. Connexins belong to the group of “gap junction” proteins, which form connexons, each consisting of six connexin molecules. These are responsible for the exchange of ions and smaller molecules between neighboring cells. Mutational analysis in genes *GJB2* and *GJB6* was brought by direct sequencing of the coding exons including the intron transitions. Here we show in the participating extended family a homozygous mutation c.506G>A, (TGC>TAC) p.Cys169Tyr, in the *GJB2* gene, which could be proven for the first time and led to nonsyndromal severe hearing impairment in the afflicted patients. The mutation is located in the EC1/EC2 interaction complex of the gap junction connexon 26 complex and interrupts the K^+^ circulation and therefore the ion homeostasis in the inner ear. The homozygous mutation p.Cys169Tyr identified here provides a novel insight into the structure-function relationship of the gap junction complex connexin/connexon 26.

## 1. Introduction

Severe prelingual sensorineural hearing impairment is diagnosed in about 2 of 1000 neonates. Nearly 50% of these cases are genetic in origin. Two-thirds of the cases are non-syndromal. 80% of the genetically caused hearing losses follow autosomal recessive transmission [1]. To date, about 140 genetic loci have been identified in connection with hearing loss, of which 49 genes have been identified and characterized (connexin-deafness homepage). 50% of all autosomal recessive inherited hearing losses show mutations in the *GJB2* gene (MIM 121011) [2]. This gene consists of two exons and is localized in tandem orientation with the *GJB6* gene (MIM 604418) at genetic locus DFNB1 A/B (MIM 220290, MIM 612645) on chromosome 13q12. Six connexins form a molecular complex, a so-called connexon, which is localized in the cell membrane and, as a “gap junction” with the corresponding connexon of neighboring cells, enables the exchange of metabolites and potassium. This electrolyte exchange is of decisive importance for the electrical potential in the cochlea [3]. To date, more than 91 different mutations in the *GJB2* gene have been proven in connection with hearing loss (connexin-deafness homepage, which lists, however, only mutations characterized until 2003). A recently published article by Hilgert et al. [4] reports that about 220 mutations in the *GJB2* gene have been described worldwide. Among the most frequently occurring mutations in the *GJB2* gene are 30/35delG, 167delT, 235delC, L90P, E47X, and M34delT [5–7]. Three large deletions have also been characterized thus far, involving part of the *GJB6* gene and the associated chromosomal downstream region, which also lead to serious non-syndromal hearing impairment.

Here we describe a novel homozygotic missense mutation in the *GJB2* gene and characterize its influence on the tertiary structure of the connexon-connexon interaction domain of the connexin 26 protein, which led to non-syndromal prelingual deafness in an extended consanguineous Arabian family from the Middle East.

## 2. Methods

### 2.1. Patient 1

**Figure 1:**
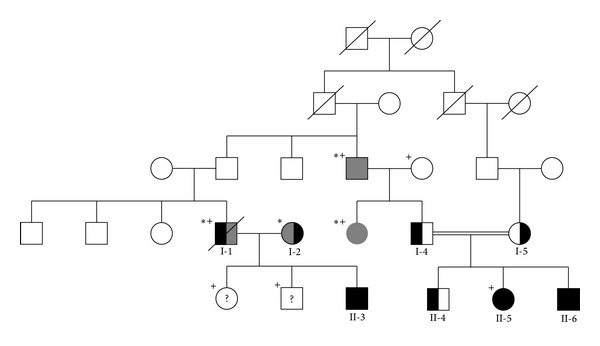
Pedigree of the family: patients are carriers of the homozygotic mutation (II-3 and II-6) (black); parents of the patients are each carrier of the heterozygotic mutation (I-2, I-4, and I-5) (half black). (*) Members of the family are also hearing impaired, but the clinical course indicates other causes (grey and half grey); (^+^) no genetic material was available; (?) no information available.

The patient, a boy, was brought to our department at the age of 14 months with suspected congenital high-grade deafness on both sides. At the age of 6 months, he had been fitted with hearing aids on both sides, which he did not tolerate. The child occasionally reacted to very close, loud noises; he was clearly face oriented in communication. There was no clinical evidence of a syndromal disease, and the pregnancy was normal. As part of preop evaluation for cochlear implantation, electrocochleography was performed on both sides after adenotomy and paracentesis. The compound action potentials were negative, and cochlear microphonics could be recorded on both sides starting at 110 dBHL. No potentials could be recorded in brainstem electric response audiometry (BERA). Hearing loss has also been found in the sister and paternal grandfather. There is no clinical evidence of a syndromal disease (Figure 1).

### 2.2. Patient 2

The patient was brought to our department at the age of 13 months with suspected congenital deafness. The patient did not react to sounds; he vocalized and followed attentively with his eyes. There were no previous diseases such as meningitis or recurrent otitides. The ENT examination revealed a nonirritative tympanic membrane on both sides, with no evidence of middle ear effusion. In the pedaudiological examination with hearing aids, he perceived the offered tones only in the deep-tone frequency range between 75 and 100 dB; no reactions could be recorded on the left side. Preevaluation for cochlear implantation was then performed. The electrocochleography revealed negative compound action potentials on both sides; cochlear microphonics could be recorded on both sides starting at 110 dBHL. Brainstem electric response audiometry (BERA) found no potentials on either side. The father and mother are both deaf. There is also familiar deafness in an uncle, a great-uncle, and 2 cousins in the father's family (Figure 1).

To rule out malformations, radiological examination of the skull was performed on both patients with CT and MRT scans. Examination showed nothing conspicuous; both the bony structures of the petrous bone and the internal auditory canal were without pathological findings (Figure 1). Subsequently, serious prelingual, bilateral impairment of sound perception (non-syndromal deafness) was diagnosed in both cases. Both patients underwent cochlear implantation (Nucleus Contour advanced electrode Cochlear Ltd., Sydney, Australia) at the age of 14 (patient II-3) and 13 (patient II-6) months, respectively.

### 2.3. Gene/Mutation Analysis

The Ethics Committee of the University of Freiburg approved this project (no. 161/02-07/2003/Birkenhäger). Genomic DNA was extracted from peripheral blood leukocytes of the patients (II-3, II-6), the mother (I-2) of patient II-3, and the parents (I-4, I-5) of patient II-6 (Figure 1), using standard methods (Qiagen). Primer and PCR conditions were selected according to procedures optimized previously for sequence analysis of the coding exon of the *GJB2* gene, including the intron transitions and deletion analysis of the *GJB6* gene [8]. Sequencing of the PCR products was done with standard procedures and analyzed in an automated DNA sequencer Amersham MegaBACE 500 (Amersham Biosciences).

## 3. Results

**Figure 2:**
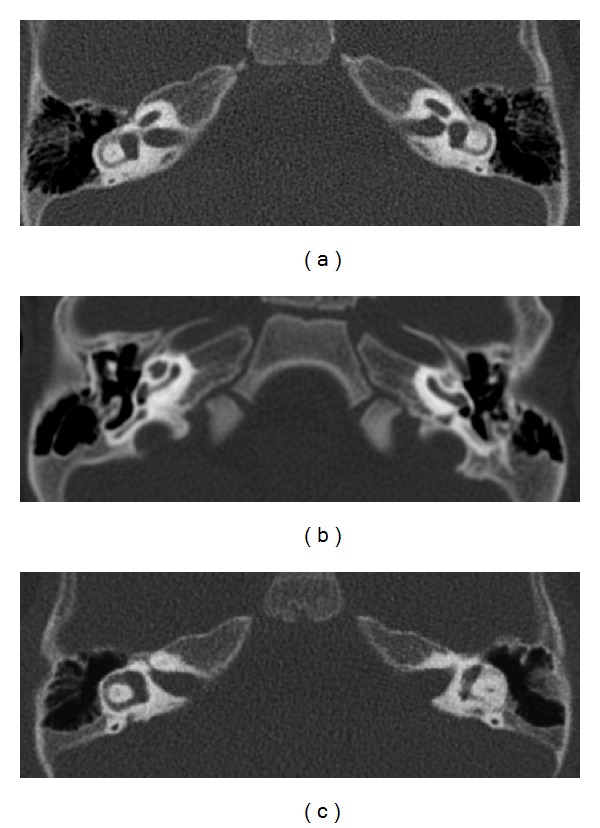
High-resolution computed tomography of the inner-ear structures of the patients: (a) II-3 and (b) II-5. There is no evidence of malformations of the cochlea or the petrous bone; (c) control image.

High-resolution computed tomography of the petrous bone revealed no morphological anomalies in the sense of malformation of the cochlea or the vestibular system in either case as the cause of prelingual non-syndromal hearing impairment (Figure 2).

**Figure 3:**
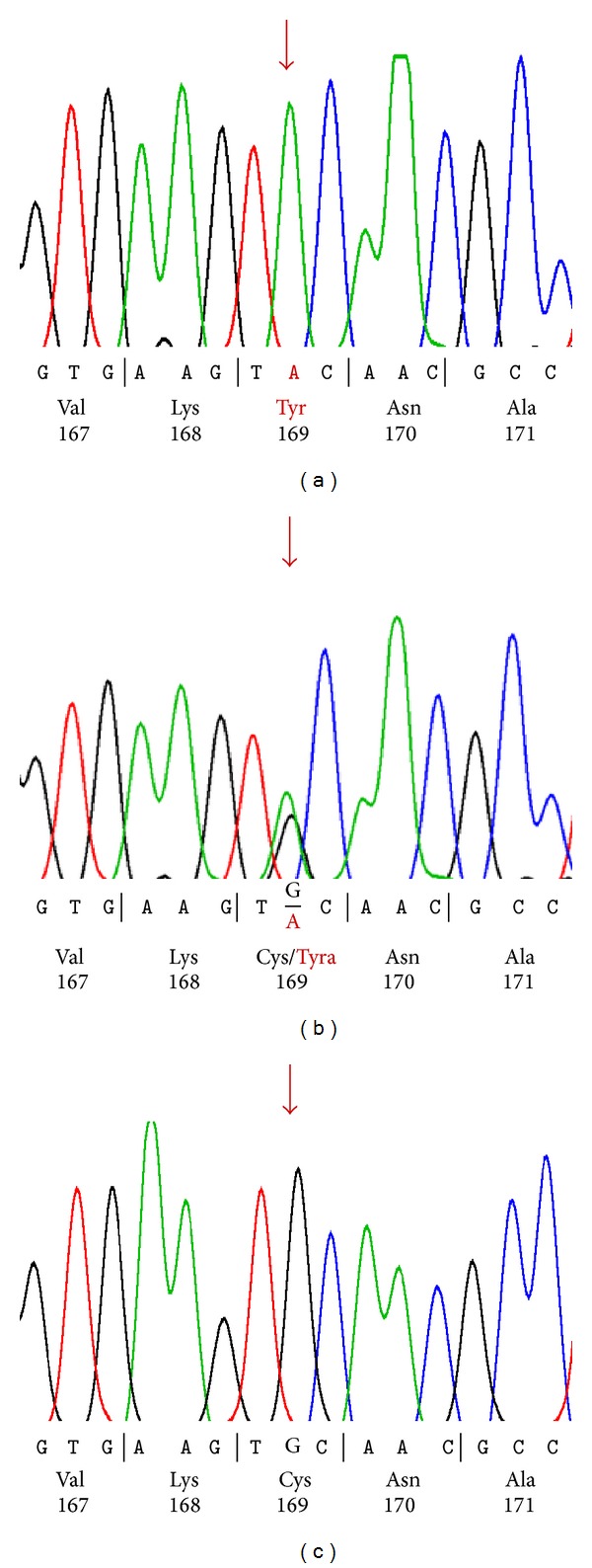
Electrogram of the mutation; (a) homozygote (c.506G>A, (TGC>TAC), Cys169Tyr) in patients (II-3, II-6); (b) heterozygote in the parents (I-2, I-4, and I-5); (c) control.

Sequence analyses of the coding exons and the intron transitions of genes *GJB2 *and *GJB6* and deletion analyses showed the same homozygotic mutation in both patients (Figure 3(a)). The mutations c.506G>A, (TGC>TAC), Cys169Tyr in the *GJB2* gene were identified in both patients. This mutation was proven heterozygous in the parents (I-2, I-4, and I-5) of the patients (Figure 3(b)). No mutation was identified in the *GJB6* gene; the known deletions were also not detected in the *GJB6* gene.

Novel missense mutations can be evaluated for possible pathogenic protein effects by prediction tools such SIFT and PolyPhen [9]. The PolyPhen [http://genetics.bwh.harvard.edu/pph2/] and SIFT [http://sift-bioinformatics.soft112.com/] prediction tools offer an in silico mechanism to investigate the potential pathogenicity of novel missense variations. When analyzing the mutation using the PolyPhen and SIFT tools, it is predicted that the new mutation p.Cys169Tyr probably changed the conformation of the connexin-26 protein. Both tools describe the fact that a change in the amino acid leads to a loss of function of the connexin 26 protein. A substitution of a tyrosine for the highly conserved cysteine changes the three-dimensional arrangement of the EC1 and EC2 subdomain, in the intracytoplasm domain of the protein complex of connexin 26, leading to a defective protein and associated deafness. This leads to a change in the extracellular domain, so that the interaction between connexon complexes is no longer possible, thus interrupting the potassium cycle in the inner ear, and ultimately the ion homeostasis can no longer be maintained.

## 4. Discussion

The causes of a non-syndromal prelingual inner-ear hearing impairment or deafness cannot always be unequivocally diagnosed at the molecular level, since more than 140 different genes and genetic loci involved in the development and function of hearing are currently under discussion (Hereditary Hearing Loss Homepage). Since gene *GJB2 *was identified and characterized, it could be shown that defects, that is, mutations, are present in this gene in more than half of all cases. To date, about 220 recessive mutations have been described [4]. Nine dominant mutations have also been identified, which are usually associated with skin diseases, such as Keratitis-Ichthyosis-Deafness Syndrome (MIM 148210) [10], Vohwinkel's Syndrome (MIN124500) [11], and palmoplantar hyperkeratosis (MIN148350) [12]. Interestingly, these are arranged exclusively in the highly-preserved CNX domain of the connexin 26 gene between amino acids 42 and 75 in the protein. By contrast, only deletions have been found in the *GJB6 *gene to date [13]. Connexins belong to a group of integral membrane proteins, of which six oligomerize and form intercellular canals, that is, “gap junction” complexes. These canals enable an exchange of low-molecular metabolites <1-2 kD, signal molecules, and ions between neighboring cells. These “gap junction” protein complexes have thus far been identified in various vertebrate cell types.

Gap junctions play important roles in different biological processes. Gap junction channels are formed by the interaction of two hemichannels, connexons, each of which is composed of six connexin molecules surrounding the central pore. In the human system, 21 different connexins have been characterized, each has distinct physiological activities. Electrophysiological studies have demonstrated that gap junctions have multiple gating mechanisms. Gap junctions can be gated by membrane voltage and by chemical factors [14]. A variety of mutations of connexin genes have been shown to be associated with a wide range of inherited diseases, for example, deafness, skin diseases, developmental abnormalities, and so forth [15, 16]. X-ray crystallography of the connexin 26 has provided structural details. Connexin 26 contains four transmembrane helices (TM1–TM4), two extracellular loops (E1, E2), and an N-terminal helix [15]. The E1 and E2 domains compose together the extracellular complex of the connexon hemichannel; this complex contributes to interhemichannel interactions of the connexons. The E1 and E2 complex of one hemichannel interacts with the corresponding E1 and E2 complex of the other hemichannel. Six conserved cysteine residues form three intramolecular disulphide bonds between E1 and E2; these bonds stabilize the structure of the extracellular region. It is conspicuous that the motifs of extracellular cysteine are conserved in all human connexins. Amino acid substitutions in any of them lead to a loss of functional gap junction channels in *Xenopus* oocyte experiments. This is probably because of the structural disorder of the extracellular region [17]. Amino acid residues that contribute to inter-hemichannel interactions are highly conserved in all connexins. Mutations of the residues that contribute to inter-hemichannel interactions are associated with human diseases, as in the case for mutations of amino acid residues involved in interactions that stabilize connexin and connexon structures [18, 19]. Oshima et al. [18] analyzed the roles of cysteine 64 residue by expressing mutant connexins in insect Sf9 and HeLa cells. Residue cysteine 64 is the third cysteine in the E1 domain, and the mutation cysteine 64 results in a loss of the electric coupling activity and caused the most profound defects among all mutations examined. They suggested that the mutated cysteine 64 has a decisive influence on oligomerization and/or protein folding and plays an important role in connexon assembly [17]. The importance of disulfide bonds in the extracellular domains has also been studied in other proteins, and the substitutions of cysteine resulted in a reduced stability [18]. All these experiments suggest that cysteine forms extracellular disulfide bridges in connexin, which is important to maintain a stable connexin structure that is suitable for connexon hemichannels formation.

In the absence of functional studies, a common approach to investigate missense mutations is by multiple complementary means: a database and the literature search, an evaluation of conservations across species, family studies, and research tools such as SIFT and PolyPhen [9]. Invertebrates have similar protein complexes with the same secondary structure, but with lower sequence identity [20]. So far, 13 different connexins have been characterized. Hydropathy analyses show that all have the same transmembranal topology, four transmembranal helices, an intracellular and two extracellular loops, N- and C-termini of the protein are located in the cytoplasm. The sequence similarity among the various isoforms is about 50–80%; the transmembranal helices are particularly well preserved [21]. By contrast, the intracellular domains, as well as the N- and C-termini, are highly variable. Both extracellular domains contain three specific cysteine residuals which are highly preserved in all connexins, forming intramolecular disulfide bridges between the cysteine residuals 53 and 180, 60 and 174, and also 64 and 169 [22]. These three disulfide bridges probably stabilize a specific three-dimensional structure of the two extracellular domains in connexin 26 molecules of a connexon complex, which is prerequisite to the interaction with a connexon of a neighboring cell. The novel homozygotic mutation c.506G>A, (TGC>TAC), Cys169Tyr, described here for the first time, localized in the second extracellular domain of the connexin 26 protein, leads to an amino acid exchange at position 169 of the connexin 26 protein of cysteine to tyrosine. In addition, as derived from the structure of the connexin 26 channel at 3.5 Å resolution, recently published by Maeda et al. 2009 [23], it is clearly demonstrated that the amino acid is important for the three-dimensional structure of the extracellular domain and thus for the interaction between two connexons. The C169Y mutation occurs in a highly conserved region of the second extracellular domain and affects one of three cysteine residuals involved in the subunit disulfide bonds that are crucial for the connexon-connexon interaction [24]. Moreover, extracellular cysteines are critical for the correct folding of the protein and essential for proper correct channel function [25]. The amino acid Cys169 is involved in one of three disulfide bridges which stabilize the three-dimensional structure between the extracellular domains E1 and E2 of the protein connexin 26. Cys169Tyr is fundamental for the interaction between two connexin complexes of adjacent cells. All evidences point to the fact that the characterized amino acid exchange Cys 169Tyr is a real mutation and is not a polymorphism as described by Murgia et al. (connexin-deafness homepage).

**Figure 4:**
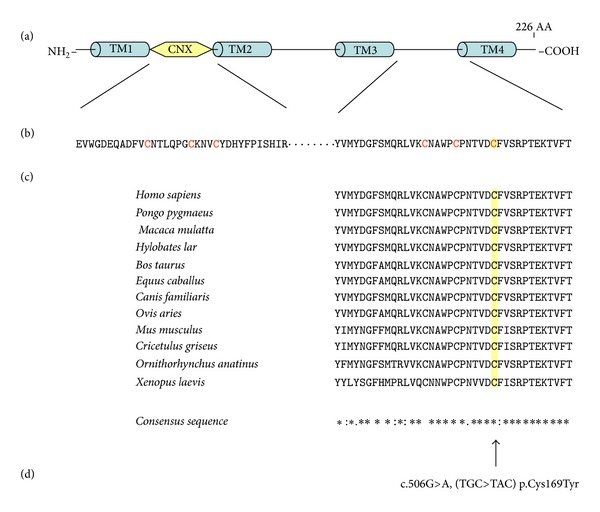
Alignment of the second extracelluar domain of the connexin 26 protein of various organisms; the identified mutation (c.506G>A, (TGC>TAC), Cys169Tyr) is strongly preserved.

**Figure 5:**
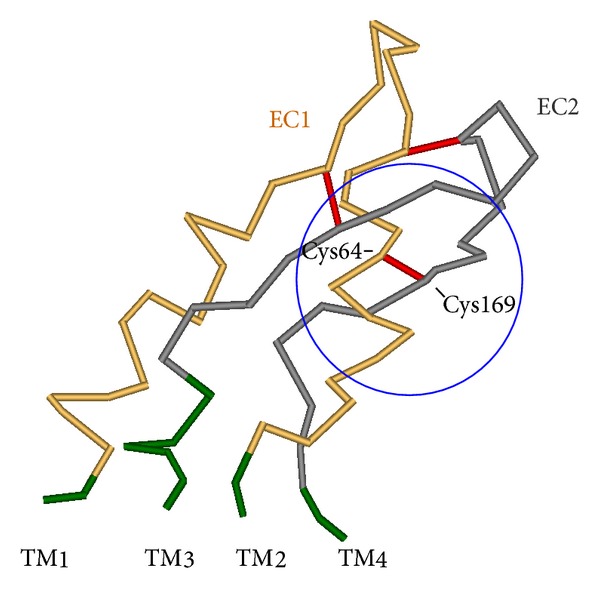
Structure analysis of the extracelluar interaction domain of a single connexin molecule; the three disulfide bridges are shown [23, 29].

Several multiple sequence alignment tools are available for evaluating the evolutionary conservation of amino acids across gene families and species. Multiple sequence alignment analyses are based largely on the assumptions that evolutionarily-conserved amino acids are more likely to be functionally important than nonconserved amino acids and substitutions involving chemically similar amino acids are less likely to be damaging than substitutions involving chemically different amino acids. However, multiple sequence alignment analyses can only provide an illustration of the degree of evolutionary conservation of an amino acid; they cannot predict with certainty the consequences of specific missense variations [26]. Ultimately, the confirmation of the pathogenicity of a sequence variation requires repeated documentation that the variation segregates with a disorder in families, detection of statistically significant differences in the frequency of the variation between large populations of case and controls, and extensive genotype-phenotype analyses. Protein alignment of the domains of twelve different organisms from *Pan troglodytes* through *Mus musculus* and *Ornithorhynchus anatinus* up to *Xenopus laevis* with humans demonstrates that the cysteine amino acid residual at position 169 is completely preserved (Figure 4). The third disulfide bridge between cysteine residuals 64 and 169 in the protein complex is destroyed by this mutation (Figure 5). The three-dimensional structure of the connexin 26 molecule and thus the superordinate connexon is probably so altered, that ultimately the interaction between connexons of neighboring cells is inhibited. These changes adversely affect the interaction between the cells, so that low-molecular substance exchange is no longer possible [27]. With respect to the functional relationships in the inner ear, the regulation of K^+^ homeostasis is no longer possible [28]. The hearing impairment of the two patients described here is probably a result of this.

## 5. Conclusion

In our study, we describe for the first time the molecular-biological analysis of genes *GJB2 *and *GJB6* in an extended family and the identification of a homozygotic mutation and its distribution within the family. Our work gives new insight into the influence of a mutation on the structure and function of connexin 26 protein. Our work indicates that, when there is evidence or presence of familiar defects, it is meaningful to examine children as early as possible for mutations in genes *GJB2* and *GJB6* in order to promptly identify non-syndromal hearing impairments and, if appropriate, initiate optimal support of speech development by means of hearing aids or cochlear implantation.
